# Multimodal Atlas of the Murine Inner Ear: From Embryo to Adult

**DOI:** 10.3389/fneur.2021.699674

**Published:** 2021-07-15

**Authors:** Jean-Paul Bryant, Vikram Chandrashekhar, Anthony J. Cappadona, Pashayar P. Lookian, Vibhu Chandrashekhar, Danielle R. Donahue, Jeeva B. Munasinghe, H. Jeffrey Kim, Alexander O. Vortmeyer, John D. Heiss, Zhengping Zhuang, Jared S. Rosenblum

**Affiliations:** ^1^Surgical Neurology Branch, National Institute of Neurological Disorders and Stroke, National Institutes of Health, Bethesda, MD, United States; ^2^Neuro-Oncology Branch, National Cancer Institute, National Institutes of Health, Bethesda, MD, United States; ^3^Center for Imaging Science, Johns Hopkins University, Baltimore, MD, United States; ^4^SmartSulis LLC, Trabuco Canyon, CA, United States; ^5^Mouse Imaging Facility, National Institute of Neurological Disorders and Stroke, Bethesda, MD, United States; ^6^Department of Otolaryngology, Georgetown University School of Medicine, Washington, DC, United States; ^7^Office of Clinical Director, National Institute on Deafness and Other Communication Disorders, Bethesda, MD, United States; ^8^Department of Pathology, Indiana University School of Medicine, Indianapolis, IN, United States

**Keywords:** Mouse, inner ear, cochlea, anatomy, development, multimodal, atlas

## Abstract

The inner ear is a complex organ housed within the petrous bone of the skull. Its intimate relationship with the brain enables the transmission of auditory and vestibular signals via cranial nerves. Development of this structure from neural crest begins *in utero* and continues into early adulthood. However, the anatomy of the murine inner ear has only been well-characterized from early embryogenesis to post-natal day 6. Inner ear and skull base development continue into the post-natal period in mice and early adulthood in humans. Traditional methods used to evaluate the inner ear in animal models, such as histologic sectioning or paint-fill and corrosion, cannot visualize this complex anatomy *in situ*. Further, as the petrous bone ossifies in the postnatal period, these traditional techniques become increasingly difficult. Advances in modern imaging, including high resolution Micro-CT and MRI, now allow for 3D visualization of the *in situ* anatomy of organs such as the inner ear. Here, we present a longitudinal atlas of the murine inner ear using high resolution *ex vivo* Micro-CT and MRI.

## Introduction

Congenital malformations of the inner ear are recognized causes of congenital hearing deficits, sensorineural hearing loss, and gait and balance disturbances (1–3). The membranous labyrinth, which is housed within the petrous bone at the skull base, serves as a common sensory organ for the cochlear and vestibular systems. The resonation of circulating endolymph within the scala media of the cochlea is detected by the organ of Corti which transmits sensory information *via* the cochlear nerve to the brainstem, where these signals are interpreted as sound. Endolymph continues from the cochlear duct to the vestibular components of the membranous labyrinth including the semicircular canals. The direction of circulating fluid within these orthogonally oriented canals is detected by the vestibular nerve and transmitted to the brainstem where it is interpreted as three-dimensional orientation. Abnormal development of the membranous labyrinth and other components of the inner ear can result in significant deficits in hearing and balance. While malformations of the inner ear may be drastic, subtle errors in the development of this complex system can lead to severe symptoms (4, 5). Thus, intimate knowledge of this anatomy is necessary for recognition of these pathologies in both mice and humans.

Many of these pathologies occur in heritable syndromes with known genetic aberrations (6, 7), emphasizing the importance of establishing mouse or other animal models. Murine inner ear anatomy has been well-studied from embryo through P6 with the greatest emphasis on embryologic days 9 through 17.5 (8, 9). However, similar to humans, the murine skull base, including the petrous bone, continues to grow until P120 (10). Therefore, to study the genetic and molecular basis of disease in these models first requires an atlas detailing inner ear anatomy from embryo to adult. To date, there is no high-resolution longitudinal imaging atlas of the inner ear in mice.

Herein, we present the anatomy of the inner ear within the intact head visualized at various time points in development including, embryo, post-natal, and adult, using high resolution *ex vivo* Micro-computed tomography (CT) and magnetic resonance imaging (MRI). We chose to evaluate the embryo at E14.5 because the components of the inner ear have reached a recognizable configuration at this time point (9). At our next time point, we evaluated the murine inner ear at post-natal day 8 (P8) because at the early post-natal stage the skull base is still undergoing development (10). Finally, we evaluated the inner ear at 5 months, which represents the adult stage, because the skull base and inner ear have completely ossified (10).

## Results

**Figure 1:**
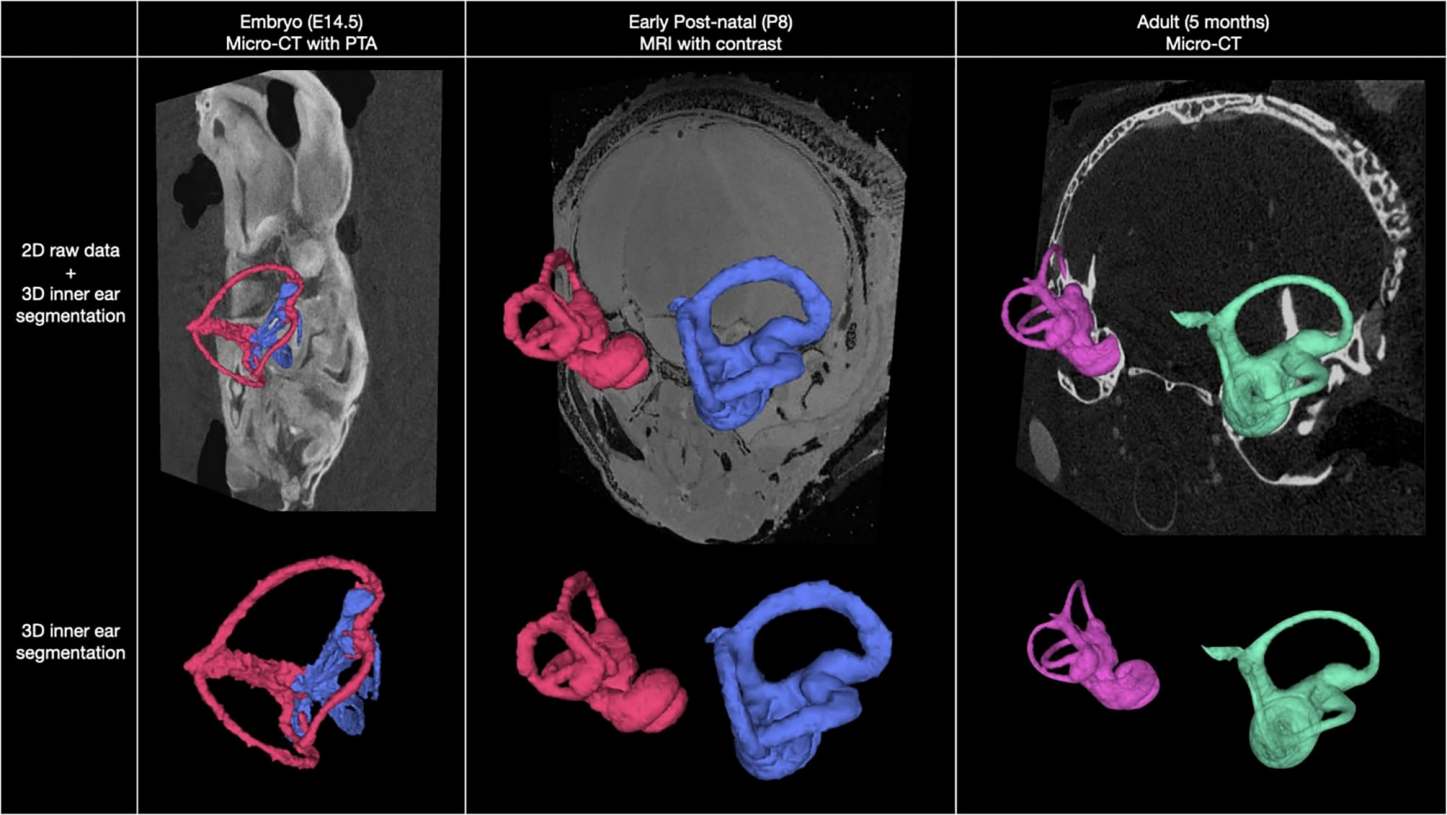
Overview of murine inner ear anatomy at three stages of development. Multimodal, 2D coronal images overlayed with 3D segmentation of the inner ear at embryo, post-natal, and adult stages (top row). Isolated 3D segmentations of the inner ear at each stage of development are shown (bottom row).

Herein we present a high-resolution, multimodal longitudinal atlas of the murine inner ear from embryo to adult. Figure 1 shows representative results of our longitudinal imaging study of murine inner ear anatomy at E14.5, P8, and adult stages. Images were acquired using Micro-CT and 14 T MRI. Using these *ex vivo* imaging modalities, we were able to evaluate the inner ear within the intact whole head in two and three dimensions at these developmental stages. In the embryo, we performed Micro-CT following immersion in phosphotungstic acid (PTA), a radio dense contrast agent (11), allowing distinction of tissues through variable X-ray attenuation that would otherwise appear homogenous on Micro-CT or MRI.

**Figure 2:**
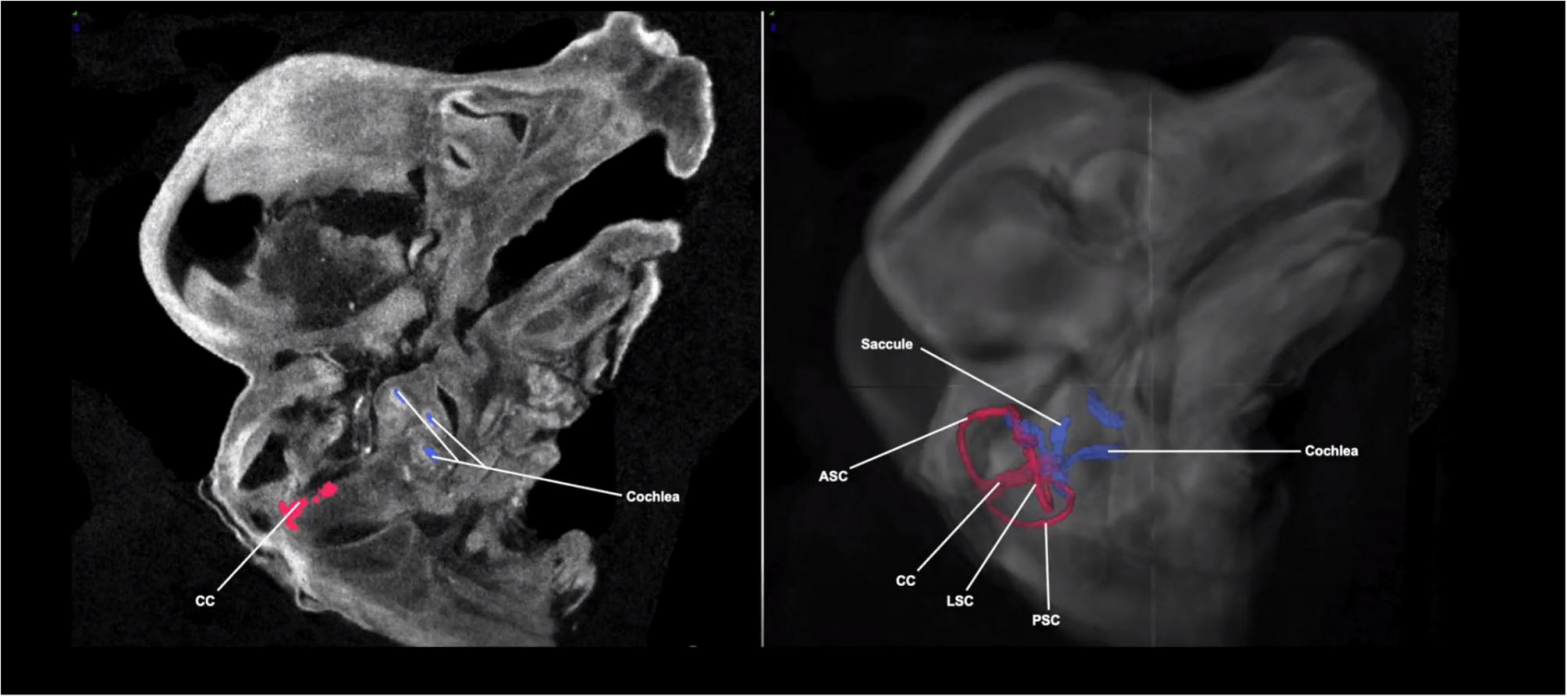
Micro-CT image with 3D segmentation of the embryological inner ear at E14.5. The anatomy of the embryonic inner ear in the sagittal plane on the 2D Micro-CT image (left). The 3D inner ear segmentation is shown overlayed on the Micro-CT image of the embryo head (right). The rudimentary semi-circular canals and common crus (red) and cochlea (blue) are shown. ASC, anterior semi-circular canal; LSC, lateral semi-circular canal; PSC, posterior semi-circular canal; CC, common crus.

Figure 2 shows a Micro-CT image with 3D segmentation of the inner ear at E14.5. A complete, annotated fly-through video of Micro-CT images with 3D segmentation of the embryological inner ear at E14.5 is displayed in Supplementary Video 1. Samples were immersed in PTA prior to image acquisition. At this stage, the primordial semi-circular canals and ampullae are visible.

**Figure 3:**
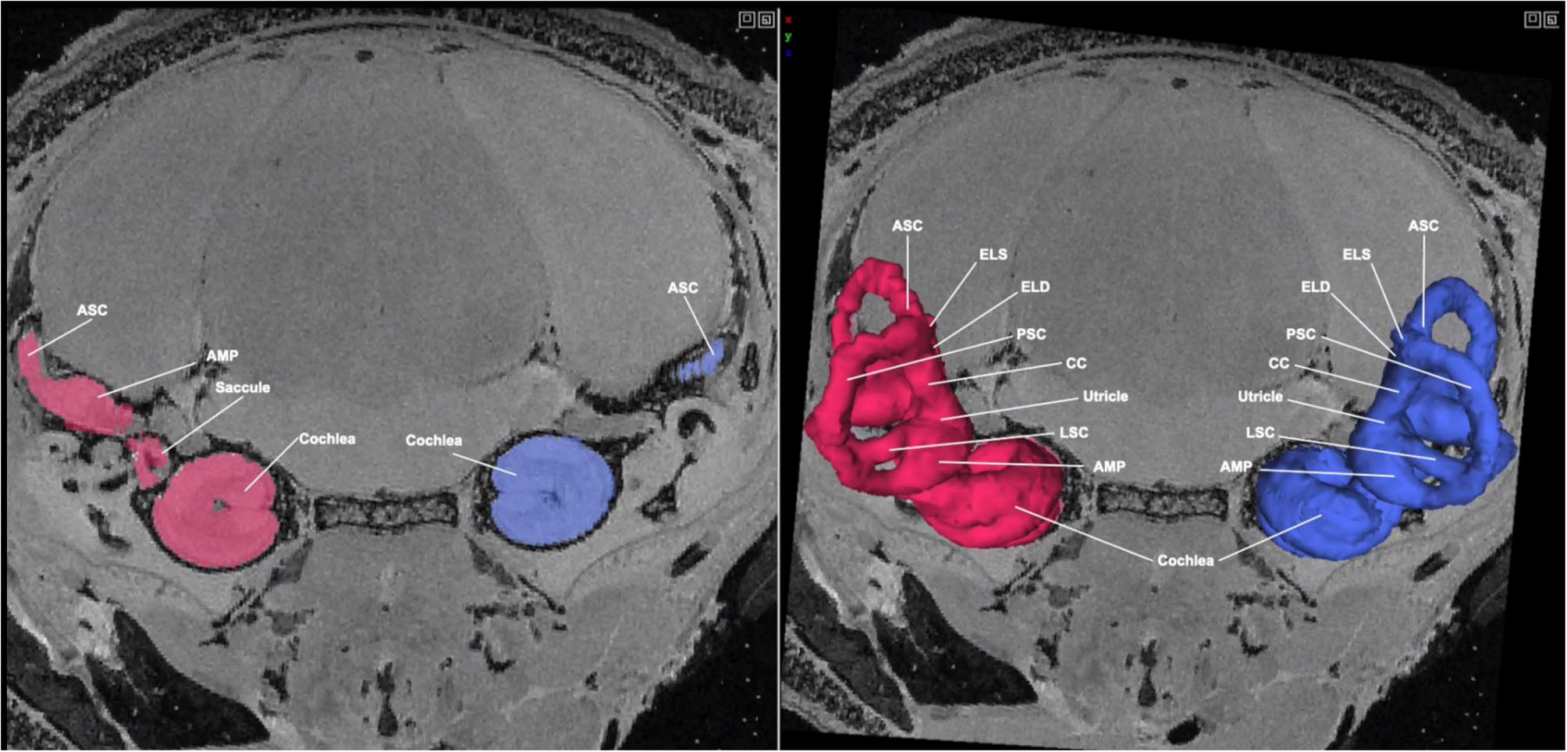
MR image with 3D segmentation of the post-natal inner ear at P8. Shown here is an MR image of the post-natal day 8 mouse head in the coronal view. The 3D segmentation is seen overlayed on the 2D image (right). The color seen in the 2D image corresponds with its respective 3D segmentation. While the inner ear structures have not fully completed maturation, they have reached their mature configuration. ASC, anterior semi-circular canal; LSC, lateral semi-circular canal; PSC, posterior semi-circular canal; CC, common crus; ELS, endolymphatic sac; AMP, ampulla.

At the next stage (P8) we evaluated, the membranous labyrinth, including the cochlea, has reached its mature configuration. Figure 3 shows an MR image with 3D segmentation of the inner ear at P8. A complete, annotated fly-through video of MR images with 3D segmentation of the post-natal inner ear at P8 is displayed in Supplementary Video 2. In addition to reaching its mature configuration, a significant increase in bone density is appreciated as the inner ear structures have undergone extensive but not complete ossification.

**Figure 4:**
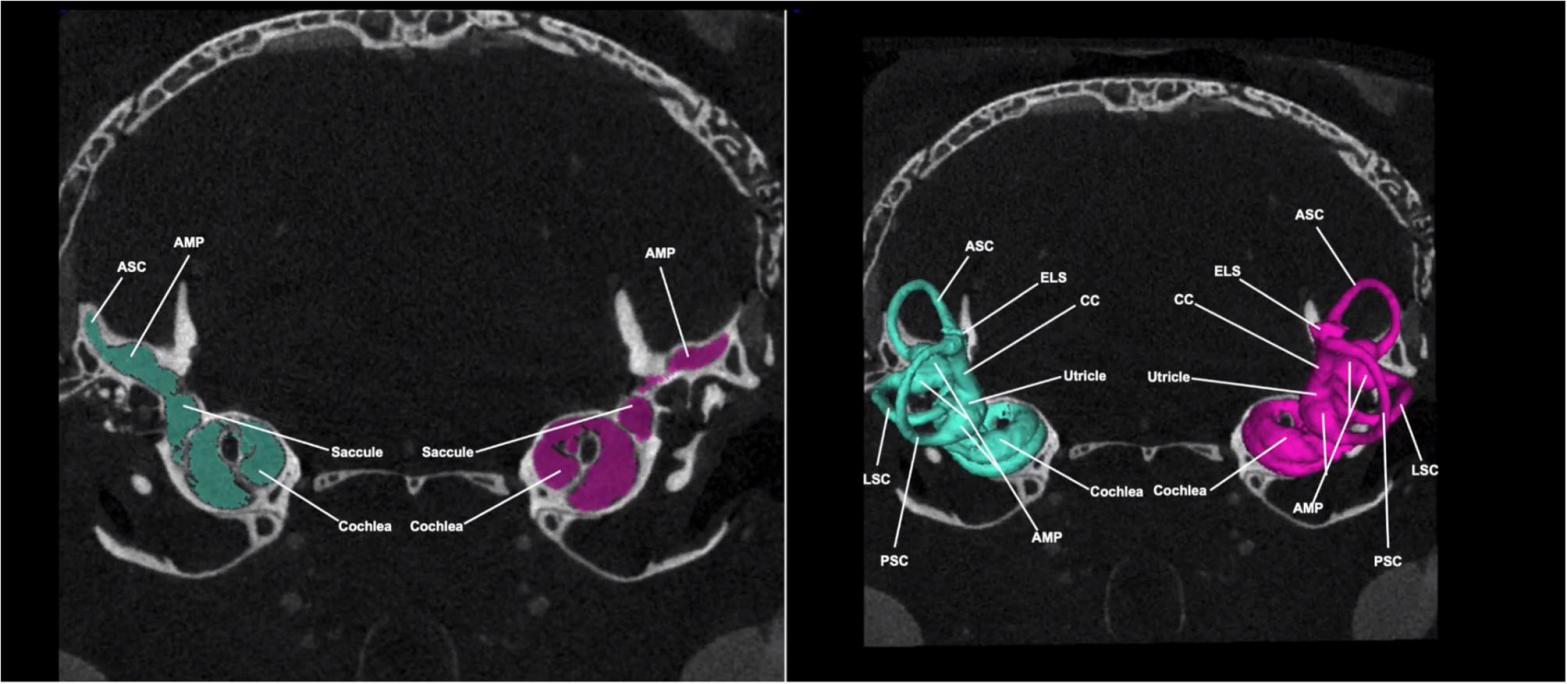
Micro-CT image with 3D segmentation of the adult inner ear. Shown here is a Micro-CT image of the adult mouse head in the coronal view. The 3D segmentation is seen overlayed on the 2D image (right). The color seen in the 2D image corresponds with its respective 3D segmentation. At this stage, inner ear structures have completed maturation and reached their final configuration. ASC, anterior semi-circular canal; LSC, lateral semi-circular canal; PSC, posterior semi-circular canal; CC, common crus; ELS, endolymphatic sac; AMP, ampulla.

Figure 4 shows a Micro-CT image with 3D segmentation of the adult inner ear. A complete, annotated fly-through video of Micro-CT images with 3D segmentation of the adult inner ear is displayed in Supplementary Video 3. At this stage, the inner ear has completed maturation and the bony labyrinth has fully ossified. The semicircular canals have grown to their full diameter and are arranged in three perpendicular planes (Figure 4). At this time point (5 months), the surrounding posterior skull base has reached its final morphology.

**Figure 5:**
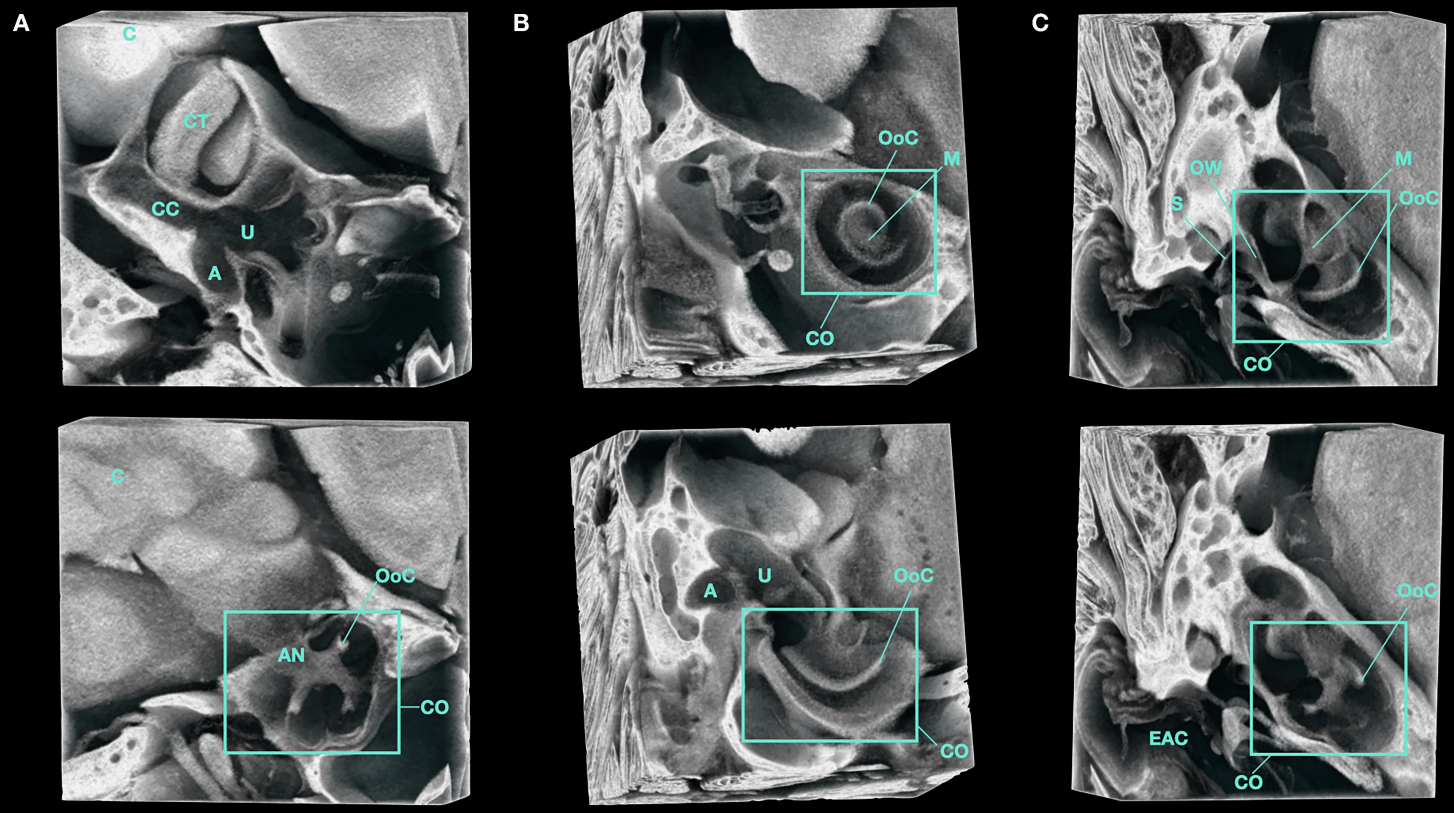
3D volume render of the inner ear of a Micro-CT imaged, PTA-immersed murine head. A representative volume of interest from the same sample is shown. **(A–C)** show orthogonal planes of the acquired data at various depths. A, Ampulla; AN, Auditory Nerve; C, Cerebellum; CT, Cerebellar Tonsil; CC, Common Crus; CO, Cochlea; EAC, External Auditory Canal; M, Modiolus; OoC, Organ of Corti; OW, Oval Window; S, Stapes; U, Utricle.

Micro-CT images of the adult inner ear following PTA immersion allows for visualization of the soft tissue structures of the membranous labyrinth within the bony labyrinth as seen in Figure 4. This high-resolution *in situ* imaging provides visualization of nearby anatomical structures such as the adjacent cerebellar tonsils as seen in Figure 5 and Supplementary Video 4. The proximity of the cerebellum to the inner ear in the mouse highlights the aforementioned embryologic relationship wherein neural crest cells migrate from the rhombomeres and envelope the early CN VIII ganglion to form central connections. These connections persist as CN VIII which can be seen entering the cochlear and vestibular canals in Supplementary Video 4. The cochlear nerve and spiral ganglion are also visible within the modiolus of the cochlea.

## Discussion

Here, we present a high-resolution, multimodal atlas of the murine inner ear in the context of its surrounding anatomy spanning from embryo to adult. Currently, a comprehensive longitudinal atlas of the *in situ*, murine inner ear throughout its development has not been reported. Traditionally, the inner ear has been studied using techniques such as histologic sectioning, paint-fill and corrosion, and whole mount (12–15). While these methods provide excellent microscopic anatomic and cellular detail, they do not provide gross anatomical information of the inner ear relative to its surrounding structures. In addition, pre-processing steps required are invasive, often damaging the underlying structure of interest. These techniques are limited to certain stages in mouse development due to difficulty of manipulating the petrous bone after ossification. Based on these considerations, we aimed to generate highly resolute images of the *in situ* murine inner ear.

We chose the imaging modality best suited to evaluate the inner ear anatomy at each stage based on the limitations of the techniques. In the adult, the significant ossification of the petrous bone would create artifacts that would limit resolution of the inner ear on MRI; thus, we chose Micro-CT. At the early post-natal stage, the bone is significantly less ossified, making visualization of the inner ear on MRI more feasible.

In this study, we demonstrate that the inner ear continues to undergo significant developmental changes throughout the postnatal period, which have not been previously described.

The membranous labyrinth arises from its embryologic precursor, a thickening in neuroectodermal tissue termed the otic placode, which is induced by rhombomeres 5 and 6 in the underlying the primitive hindbrain between embryonic day 8 and day 8.5 (12, 16). The otic placode subsequently invaginates to form the otic cup and eventually, a rudimentary otocyst by E9.5 (9). Following induction of the neurogenic domain of the otic placode, neuroblasts delaminate from the otic epithelium at the anteroventral otic cup and converge to form the neurons of the cochleovestibular (CN VIII) ganglion. The emergence of these neuroblasts occurs in close proximity to neural crest cells migrating from the hindbrain at rhombomere 4 toward the second pharyngeal arch (17). The primordial CN VIII ganglion is subsequently enveloped by this neural crest stream, forming central axonal connections with the developing hindbrain by E10.5 (9). The necessity of this complex interaction of these migrating neural crest cells with ectoderm, mesenchyme, and neuronal precursors for proper formation of the inner ear was highlighted by several lineage tracing studies. These studies demonstrated that neural crest cells give rise to the glial cells, melanocytes of the stria vascularis, and a portion of the bony labyrinth (18, 19). At approximately E16.5, the rudimentary otocyst has given rise to the recognizable structure of the membranous labyrinth. This includes a vestibular component, comprised of the maculae located within the primitive utricle and saccule, and an auditory component, consisting of the cochlear anlage. The cochlear anlage develops from the ventral portion of the rudimentary otocyst and will go on to form the mature cochlea after coiling one and three-quarter turns by E17 (12). This early embryonic period also gives rise to the endolymphatic duct and sac, which emerge as a single dorsal protrusion from the rudimentary otocyst at ~E10.5 (20). At E13.5 these structures are anatomically distinguishable (9).

At E14.5, the cochlea has begun to coil, but it has not yet reached its mature configuration of 1.75 turns. Additionally, the semicircular canals, utricle, and saccule are underdeveloped as evidenced by an apparent lack of fluid filling these structures.

At our next evaluated time point, P8, we see a more mature inner ear including a coiled cochlea and a fluid-filled membranous labyrinth within a larger petrous bone. Studies in humans have indicated that the skull base, including the petrous bone which houses the inner ear, experiences the most rapid period of growth within the first 12 months of age (21). Thus, we evaluated murine inner ear anatomy at the early post-natal stage to characterize structural changes from its embryonic form. At this stage, we see that while the membranous labyrinth has neared complete maturation, the bony labyrinth has not. The semi-circular canals are now fluid-filled spaces containing endolymph and perilymph. The endolymphatic duct and sac are easily distinguishable and have undergone significant development compared to the previous stage (E14.5). The endolymphatic sac will eventually reach its final position partially within the temporal bone and partially in its extra-osseous location within the dura of the posterior fossa (22). In addition, the opening of the tunnel of Corti and formation of the spaces of Nuel occur between P6 and P10 (23). Whole head imaging at the post-natal stage allowed us to visualize these structures within the context of the surrounding petrous and temporal bones. By P8, the membranous labyrinth is encased in a thin bony labyrinth within the otic capsule of the petrous temporal bone at the skull base. These bones will continue to develop to reach their adult configuration.

At the adult stage, the cochlear nerve and spiral ganglion are visible within the modiolus of the cochlea (Supplementary Video 4). The modiolus is a broad-based bony structure that constitutes the canonical central axis of the cochlea and is comprised of spongy bone which transmits filaments of the cochlear nerve through its perforations (1). Shortened modiolar length has been identified as important criteria in classifying congenital anomalies of the cochlea, such as Mondini dysplasia (24). Further, the Micro-CT images following PTA immersion allow for phenotypic characterization of portions of the membranous and bony labyrinth that have distinct embryologic relationships and clinical correlates to congenital inner ear malformations (5). Thus, high resolution imaging of inner ear structures can provide translatable, anatomic information in mouse or other animal models such as rats (25, 26). Further, our study can be used for comparison to existing studies using Micro-CT to evaluate developmental ear pathologies in mouse models.

Herein, we present a longitudinal anatomical atlas of the murine inner ear ranging from embryo to adult. While embryonic development of the inner ear has been previously described, a longitudinal atlas of the *in-situ* bony and membranous labyrinth has yet to be reported. To our knowledge, our atlas is the first to report high-resolution 2D and 3D representations of the intact murine inner ear at embryonic, postnatal, and adult time points. Our MRI and Micro-CT imaging techniques allowed the samples to be evaluated intact. In addition, this allowed iterative sample processing and re-imaging of the whole mouse head and preservation of important underlying anatomical structures. The methodology used in this study may prove useful for evaluating murine inner ear malformations. We have provided annotated atlases for critical embryologic, post-natal, and adult time points that can be referenced when studying relevant disease models.

## Materials and Methods

We evaluated C57BL/6 mouse inner ear anatomy at the embryologic (E14.5), post-natal (P8), and adult stages of development using Micro-CT and 14 T MRI. Post-natal and adult mice were perfused with Microfil silicate polymer (Flowtech Inc, Carver, MA) to allow for separation of vascular structures from the surrounding bone as previously described (27).

### Micro-CT

We evaluated the embryo and adult stages using high-resolution Micro-CT. Images of the polymer-casted adult mice (*n* = 5; male = 3, female = 2) fixed in 4% paraformaldehyde were acquired as previously described (27). The parameters for this adult scan using Skyscan1172 (Bruker Micro-CT, Kontich, Belgium) were as follows: nominal resolution of 13.53 μm, 0.5 mm Aluminum filter and the X-ray source biased at 65 kV and 110 μA. Six projections were averaged together every 0.4° for a 180° scan, each with an exposure time of 1,600 ms. Images of the E14.5 (*n* = 3; male = 1, female = 2) fixed in 4% paraformaldehyde and contrast-enhanced with PTA were performed on the Skyscan1272 (Bruker Micro-CT, Kontich, Belgium), with a nominal resolution of 3.02 μm, necessitated the use of 0.5 mm Aluminum and 0.038 mm Copper filters and the X-ray source biased at 90 kV and 110 μA. Ten projections were averaged together every 0.10° for a 360° scan, each with an exposure time of 2,175 ms. Images of fixed and polymer-casted murine samples were first acquired to visualize normal bony inner ear anatomy. Mouse samples were then decalcified in a 10% hydrochloric acid immersion for 72 h. To visualize soft tissue of the membranous labyrinth, samples were dehydrated in subsequent increasing ethanol concentrations and then immersed in PTA before being scanned again using Micro-CT as previously described (11).

Reconstruction was carried out with a modified Feldkamp_ii_ algorithm using the SkyScanTM NRecon software accelerated by GPU_iii_. Gaussian smoothing, ring artifact reduction, and beam hardening correction were applied (as applicable).

### MRI

MRI images were acquired of the post-natal (P8) stage (*n* = 3; male = 2, female = 1). The sample was immersed in 4% paraformaldehyde for 36 h, then 0.1% Magnevist/PBS solution for 24 h preceding MRI. The tissue was placed in 15 mm NMR sample tubes, filled with Fluorinert to match the magnetic susceptibility between the tissue and surroundings, and positioned in the scanner where the coil performance was optimized. Scans were performed using a 14 T MRI scanner equipped with a 40 mm bore scanner, capable of gradient strength of 156 G/cm. The size of the radio frequency coil was 15 mm. The imaging parameters including field of view and matrix size were maintained to achieve a constant isotropic resolution. Brain images were acquired at 50 μm isotropic resolution echo time (TE) = 5 ms, repetition time (TR) = 50 ms, flip angle = 30°. Scout images and 3D gradient echo images were acquired of the sample.

### Image Processing

To generate 3D segmentations of the bony and membranous labyrinths, we used the semi-automated active contour segmentation tool in the ITK-SNAP 3.0 software application (Researchers at University of Pennsylvania and UNC, USA) on all acquired images. The resulting segmentations were proof-read and edited when necessary using the paint brush tool in ITK-SNAP. We saved the proof-read segmentation items as NIFTI files and converted them into a mesh format compatible with Neuroglancer, an open-source, interactive volumetric data viewing tool. The 3D renders were visualized overlayed on acquired 2D images in Neuroglancer (28).

## Data Availability Statement

The raw data supporting the conclusions of this article will be made available by the authors, without undue reservation.

## Ethics Statement

The animal study was reviewed and approved by IACUC at the National Institutes of Health.

## Author Contributions

J-PB, VikC, and JR conceived the idea, developed the methodology, performed validation, performed formal analysis, curated data, visualized the data, and wrote, reviewed, and edited the original draft. AC developed the methodology, generated data, and reviewed, and edited the manuscript. PL and VibC analyzed data and reviewed and edited the manuscript. DD and JM generated data and reviewed and edited the manuscript. HK performed formal analysis, performed validation, and reviewed and edited the manuscript. AV and JH reviewed and edited the manuscript. ZZ supervised the investigation, provided resources, and reviewed and edited the manuscript. All authors contributed to the article and approved the submitted version.

## Conflict of Interest

VibC was employed by company SmartSulis LLC. The remaining authors declare that the research was conducted in the absence of any commercial or financial relationships that could be construed as a potential conflict of interest.
