# Supplementary material for: Multimodal Atlas of the Murine Inner Ear: From Embryo to Adult
Source: Front Neurol. 2021 Jul 15;12:699674. doi: 10.3389/fneur.2021.699674 (PMC8319626; doi:10.3389/fneur.2021.699674)
Supplement: Supplementary file 1 [file Table_1.DOCX]

**Supplementary Materials**

**Supplementary Figures…2-5**

**Supplementary Figures**


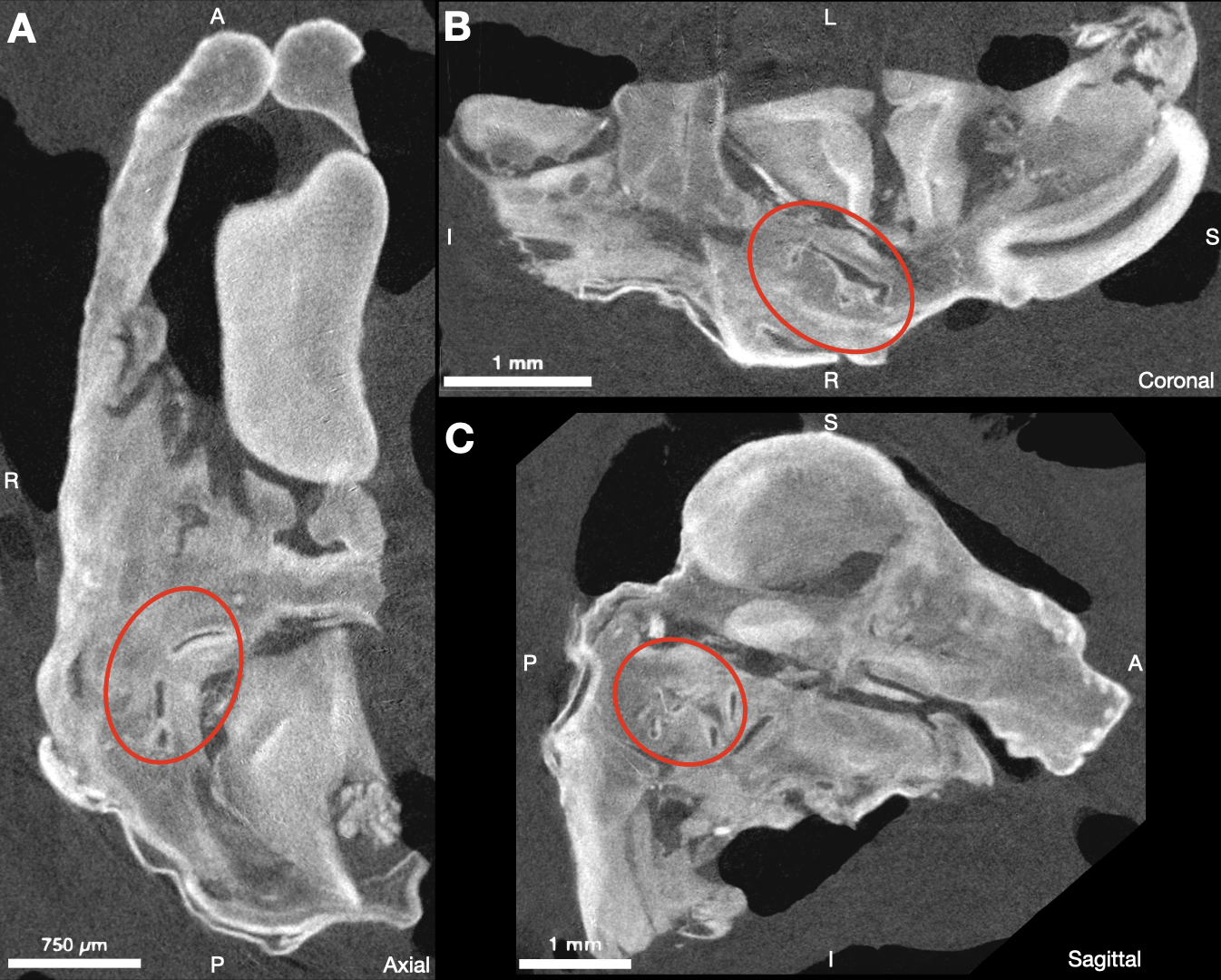
Supplementary Figure 1. **2D Micro-CT images of PTA-immersed murine embryo.** Raw 2D Micro-CT images of the half embryo head in axial (A), coronal (B), and sagittal (C) views. The developing membranous labyrinth structures are circled in red. PTA, Phosphotungstic Acid; CT, Computed Tomography.


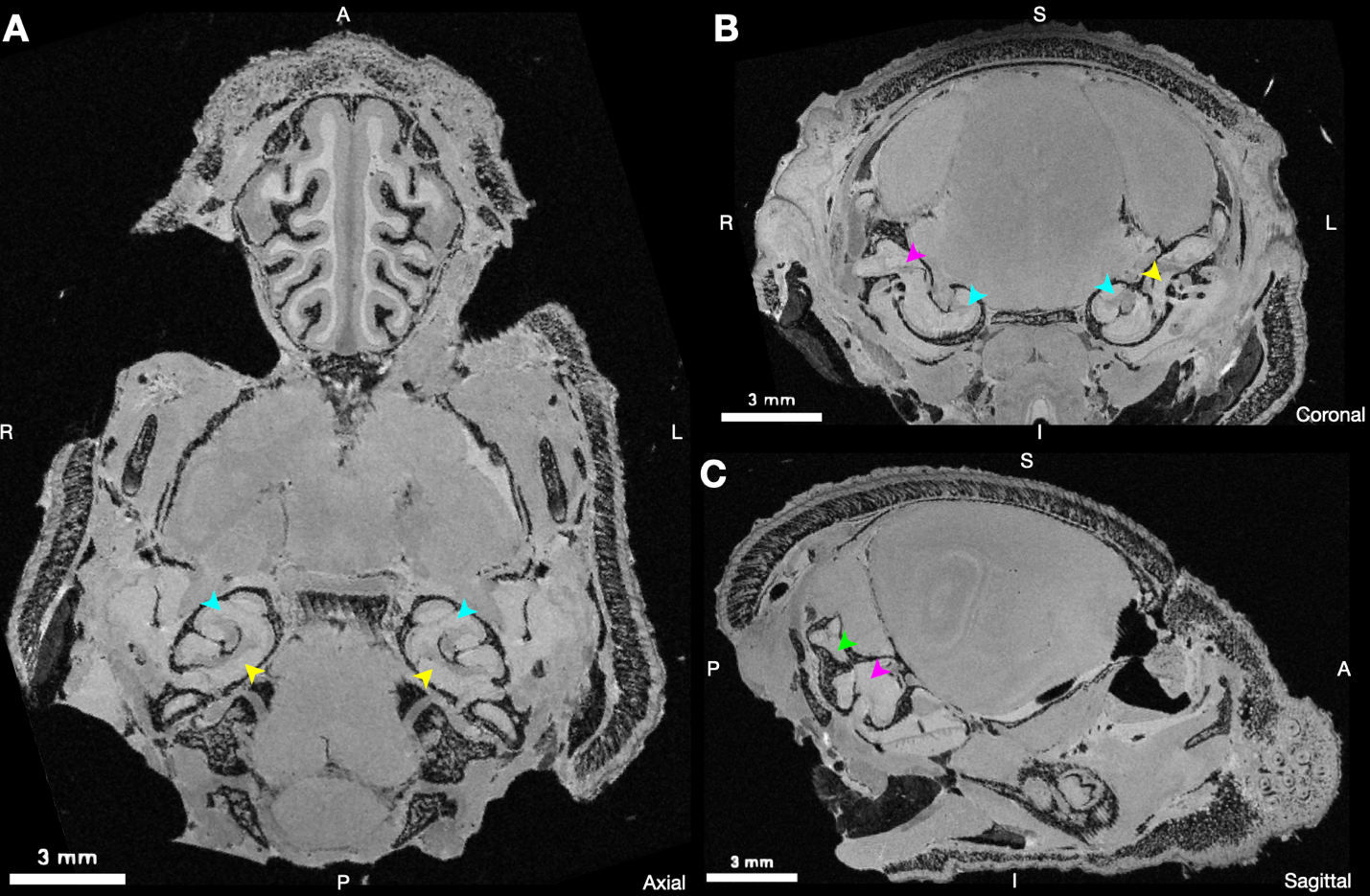
Supplementary Figure 2. **2D MRI images of contrast-enhanced murine post-natal head.** Raw 2D MRI images of the Gadolinium-immersed post-natal mouse head in axial (A), coronal (B), and sagittal (C) views. Membranous labyrinth structures are highlighted with arrowheads; the common crus is green, cochlea is cyan, cochlear duct is yellow, and utricle is magenta. MRI, Magnetic Resonance Imaging.


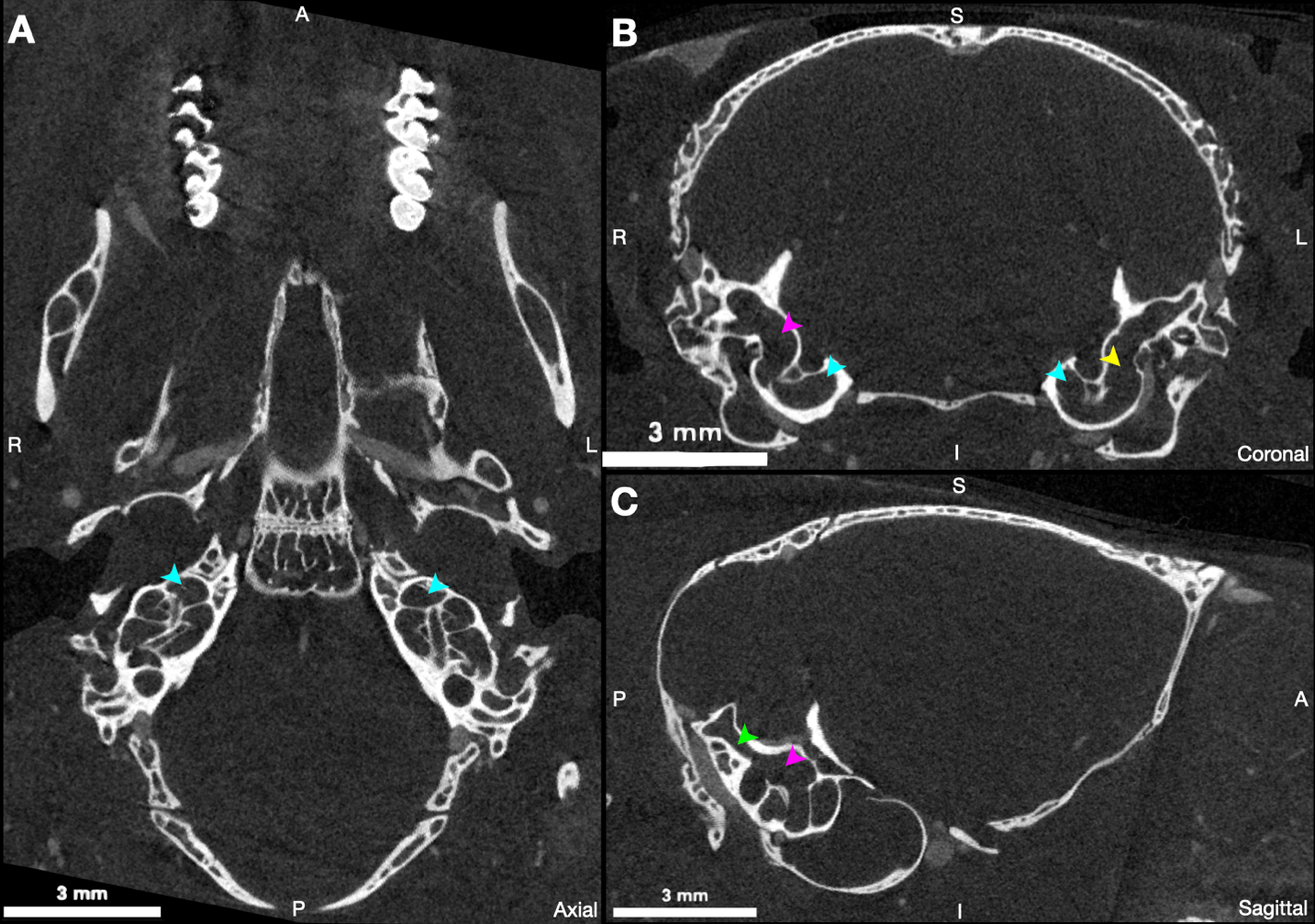
Supplementary Figure 3. **2D Micro-CT images of murine post-natal head.** Raw 2D Micro-CT images of the polymer-perfused murine adult head in axial (A), coronal (B), and sagittal (C) views. Membranous labyrinth structures are highlighted with arrowheads; the common crus is green, cochlea is cyan, cochlear duct is yellow, and utricle is magenta. CT, Computed Tomography.
